# Profiling olfactory stem cells from living patients identifies miRNAs relevant for autism pathophysiology

**DOI:** 10.1186/s13229-015-0064-6

**Published:** 2016-01-08

**Authors:** Lam Son Nguyen, Marylin Lepleux, Mélanie Makhlouf, Christelle Martin, Julien Fregeac, Karine Siquier-Pernet, Anne Philippe, François Feron, Bruno Gepner, Claire Rougeulle, Yann Humeau, Laurence Colleaux

**Affiliations:** INSERM UMR 1163, Laboratory of Molecular and pathophysiological bases of cognitive disorders, Paris Descartes - Sorbonne Paris Cité University, Imagine Institute, Necker-Enfants Malades Hospital, 24 boulevard du Montparnasse, 75015 Paris, France; Synapse in Cognition Laboratory, Institut Interdisciplinaire de NeuroSciences, Centre de génomique fonctionnelle, UMR 5297 CNRS - Université de Bordeaux, 146 rue Léo Saignat, 33077 Bordeaux, France; Epigénétique et Destin Cellulaire, Université Paris Diderot, UMR 7216, 75205 Paris, France; Aix Marseille Université, NICN, CNRS UMR 7259, 13344 Marseille, France

**Keywords:** Autism spectrum disorders, MicroRNA, Neuron, Astrocyte, Olfactory mucosa stem cells

## Abstract

**Background:**

Autism spectrum disorders (ASD) are a group of neurodevelopmental disorders caused by the interaction between genetic vulnerability and environmental factors. MicroRNAs (miRNAs) are key posttranscriptional regulators involved in multiple aspects of brain development and function. Previous studies have investigated miRNAs expression in ASD using non-neural cells like lymphoblastoid cell lines (LCL) or postmortem tissues. However, the relevance of LCLs is questionable in the context of a neurodevelopmental disorder, and the impact of the cause of death and/or post-death handling of tissue likely contributes to the variations observed between studies on brain samples.

**Methods:**

miRNA profiling using TLDA high-throughput real-time qPCR was performed on miRNAs extracted from olfactory mucosal stem cells (OMSCs) biopsied from eight patients and six controls. This tissue is considered as a closer tissue to neural stem cells that could be sampled in living patients and was never investigated for such a purpose before. Real-time PCR was used to validate a set of differentially expressed miRNAs, and bioinformatics analysis determined common pathways and gene targets. Luciferase assays and real-time PCR analysis were used to evaluate the effect of miRNAs misregulation on the expression and translation of several autism-related transcripts. Viral vector-mediated expression was used to evaluate the impact of miRNAs deregulation on neuronal or glial cells functions.

**Results:**

We identified a signature of four miRNAs (*miR-146a*, *miR-221*, *miR-654-5p*, and *miR-656*) commonly deregulated in ASD. This signature is conserved in primary skin fibroblasts and may allow discriminating between ASD and intellectual disability samples. Putative target genes of the differentially expressed miRNAs were enriched for pathways previously associated to ASD, and altered levels of neuronal transcripts targeted by *miR-146a*, *miR-221*, and *miR-656* were observed in patients’ cells. In the mouse brain, *miR-146a*, and *miR-221* display strong neuronal expression in regions important for high cognitive functions, and we demonstrated that reproducing abnormal *miR-146a* expression in mouse primary cell cultures leads to impaired neuronal dendritic arborization and increased astrocyte glutamate uptake capacities.

**Conclusions:**

While independent replication experiments are needed to clarify whether these four miRNAS could serve as early biomarkers of ASD, these findings may have important diagnostic implications. They also provide mechanistic connection between miRNA dysregulation and ASD pathophysiology and may open up new opportunities for therapeutic.

**Electronic supplementary material:**

The online version of this article (doi:10.1186/s13229-015-0064-6) contains supplementary material, which is available to authorized users.

## Background

Autism spectrum disorders (ASD) represent a group of childhood neurodevelopmental disorders characterized by deficits in verbal communication and social interaction and restricted and repetitive patterns of interests and behaviors. These conditions are often considered as “connectopathies” or “synaptopathies,” whereby neuronal circuits are mis-wired or dysfunctional. Much progress has been made in unravelling the genetics of ASD with large copy number variation [1] and de novo mutations in a subset of genes [2] as well as inherited mutations being associated with ASD [3]. Yet, the current consensus agrees that majority of ASD cases are caused by complex interaction of multiple genetic and environmental risk factors. Orchestrated by environmental factors, epigenetic modifications, such as DNA methylation, covalent modification of histones, or the activation or silencing of genes by microRNAs (miRNAs), may drive genetically predisposed individuals to develop autism and represent the crossroads between the environment and genetics in ASD. Accordingly, gene expression profiling of monozygotic twins discordant in diagnosis of autism has identified differentially expressed, neurologically relevant genes [4]. Similarly, abnormal DNA methylation patterns have been detected at several candidate genes in ASD patients [5].

miRNAs play key roles in neuronal development, synapse formation, and fine-tuning of genes underlying synaptic plasticity and memory formation [6]. Several studies have identified aberrant miRNA expression in a wide range of neurological diseases [7, 8], but a comprehensive understanding of miRNA networks deregulated in ASD has not been achieved. The expression of miRNAs in lymphoblastoid cell cultures of ASD patients has already been explored [9–12], but the relevance of this cell type is questionable in the context of a neurodevelopmental disorder. One study has identified deregulated miRNAs in the cerebellum of ASD patients [12] and another investigated the superior temporal sulcus and auditory cortex [13], but it raises the issue of the investigated brain regions and the impact of cause of death and/or post-death handling of tissue on gene expression.

To overcome these limitations, we used olfactory mucosal stem cells (OMSCs) biopsied from living ASD patients and controls. OMSCs exhibit characteristics of ecto-mesenchymal stem cell and maintain neurogenic ability [14, 15]; they could be considered as precursor of neuronal and glia-like cells. These characteristics make OMSC a very relevant model for transcriptomic studies with great potential to identify genes and pathways relevant for neurodevelopmental disorders. Indeed, OMSC has been used successfully to study the pathology of schizophrenia [16], Rett syndrome [17], and Parkinson’s disease [18] in which they displayed disease-specific alterations in gene expression and cellular functions (for review, see [19]).

Here, using OMSC, we report a signature of miRNA commonly deregulated in ASD. We show that this signature is conserved in primary skin fibroblast. We demonstrate that these miRNAs have essential roles in brain function through their regulation of neuronal specific genes and provide evidence that deregulation of their expression alter the development of primary neurons and the function of glial cells. Our results demonstrate the importance of the miRNA circuitry in ASD, opening up opportunities for the development of early diagnostic test and potential treatment.

## Methods

### Ethic statements

Human samples were obtained with informed consent of the patients, and studies were carried out under a protocol that was approved by the ethic committees of the Hôpital Necker, Paris, and the Montperrin Hôpital, Aix en Provence (CPP Marseille2). The experiments conformed to the principles set out in the WMA Declaration of Helsinki and the Department of Health and Human Services Belmont Report. All the biological collections (blood, nucleic acid, tissues) are kept securely in one place, and their management and the quality of their preservation follow the French regulations and ethical recommendations. Primary cortical neurons were extracted from mouse embryos (E15.5) following protocols approved by the local ethical committee of the Interdisciplinary Institute of Neuroscience of Bordeaux accordingly to the European Communities Council Directive (86/809/EEC).

### Patients’ information

The patients included in this study have been clinically diagnosed as having ASD according to the criteria of DSM-V. Full clinical description of these patients has already been reported [20]. We include here a brief clinical description of the patients (see Additional file 1: Table S1). To investigate the genetic etiology, we extracted DNAs from their OMSCs and subjected to array comparative genomic hybridization and whole exome sequencing according to published protocol. One patient (A3) carries two variants leading to premature translation termination in *SCN2A* and *FAT1*, which are both recurrent non-fully penetrant ASD genes (see Additional file 1: Tables S2 and S3); two patients (A1 and A10) were found to carry deletions at genomic regions 22q13.3 and 15q13.2–q13.3, respectively, which have been found to be associated with ASD (see Additional file 1: Table S3). The genetic etiology of the remaining patients remained inconclusive.

### Maintaining of OMSC, human primary fibroblast, and HEK293T

OMSC was biopsied and cultivated according to published protocol [20]. OMSC was maintained using Dulbecco’s modified Eagle’s medium (DMEM)/F-12 GlutaMAX™ (Life Technologies) supplemented with Penicillin-Streptomycin (100 U/ml) (Life Technologies) and 10 % fetal bovine serum (FBS). Human primary fibroblasts were extracted from skin punches with informed consents and maintained in RPMI 1640 Medium GlutaMAX™ (Life Technologies) supplemented with Penicillin-Streptomycin (100 U/ml) (Life Technologies) and 10 % FBS. HEK293T cells were maintained in DMEM High-Glucose Medium GlutaMAX™ (Life Technologies) supplemented with Penicillin-Streptomycin (100 U/ml) (Life Technologies) and 10 % FBS. Both cell types were passaged first by detaching using 0.05 % Trypsin-EDTA (1×) Phenol Red (Life Technologies) and replated in desired concentration.

### Expression profiling of miRNA

miRNAs were extracted from frozen pellets using mirVanaTM miRNA isolation kit (Life Technologies) according to manufacturer’s instructions. Concentration was measured using Nanodrop 2000 (Thermo Scientific). During the discovery phase, miRNA profiling was performed across a two-card set of TaqMan® MicroRNA Arrays (arrays A and B) (Life Technologies) for a total of 667 unique assays specific to human miRNAs (Sanger miRBase v10). Analyses were done in technical triplicates and used two different miRNA extractions from two pellets. For validation, 24 miRNAs that were found either significantly deregulated in the first round or whose assays failed and the 7 reference miRNAs were assessed (see Additional file 1: Figure S1). Raw data (see Additional file 2) were analyzed using both GenEX and RealTime StatMiner® software. Both integrate quality controls, selection of best endogenous controls, clustering, differential expression, and two-way ANOVA analysis. The samples included in miRNA profiling were A2, A3, A5, A6, A7, A9, A10, and A11 vs. C1, C4, C5, C6, C7, and C10. The miRNA profiling and analyses were performed as paid service by the IMAGIF platform for high-throughput quantitative PCR, ICSN CNRS, Gif-sur-Yvette, France. Expression profiles of *miR-146a*, *miR-221*, *miR-654-5p*, and *miR-656* were assessed using Taqman assays on Fluidigm 48.48 array in technical triplicates; for fibroblast samples, two different miRNA extractions from consecutive passages were analyzed and six miRNAs were used as reference miRNAs (see Additional file 1: Figure S2 and Additional file 2 for raw data); for peripheral blood mononuclear cells (PBMC), only one extraction was analyzed and seven miRNAs were used as reference miRNAs (see Additional file 1: Figure S3 and Additional file 2 for raw data). Sample preparation was done according to the manufacturer’s Protocol for Creating Custom RT and Preamplification Pools using Taqman® MicroRNA Assays (Life Technologies). This step was performed as paid service by Platform qPCR-HD-GPC, Ecole Normale Supérieure, Paris, France.

### miRNA target prediction and pathway analysis

Target prediction was performed using mir-DIP which integrates predictions from multiple software. Only targets predicted by at least three different programs were included in pathway analysis. Gene ontology enrichment of predicted miRNA targets (~1000 targets per miRNA) was performed using ingenuity pathway analysis (IPA).

### Quantitative real-time polymerase chain reaction

Total RNA was extracted using TRIzol reagent (Life Technologies) and RNeasy Mini Kit (QIAGEN) with DNase I treatment step (QIAGEN). cDNA was reversed transcribed from extracted RNA using SuperScript® II Reverse Transcriptase (Life Technologies). Gene expression was measured using SYBR Green Power Mix (Life Technologies) using primers specific for each gene. Additional file 1: Table S4 contains the sequences of all primers used in this study. Relative standard curve method was employed to calculate relative gene expression. Reactions were performed using the Step One Plus Real-Time PCR system (Applied Biosystems). Expression values were taken from mean of triplicates.

### Cloning the 3′UTR of *GRIA3*, *KCNK2*, and *MAP2*

The entire or parts of the 3′ untranslated region (UTR) of *GRIA3* (~2000 bp), *KCNK2* (~1300 bp), and *MAP2* (~1300 bp) were amplified using specific primers (Additional file 1: Table S4), subcloned into TOPO 3.1 vector using TOPO® TA Cloning® Kit (Life Technologies) and transformed into One Shot® TOP10 Chemically Competent E. coli (Life Technologies) by heat shock method. Plasmid was extracted using PureYieldTM Plasmid MiniPrep System (Promega) and digested with XhoI and NotI-HF (New England Biolabs). Digested fragment was gel purified once more then cloned into cut psiCheck2 plasmid (Promega) using T4 DNA Ligase (New England Biolabs). For higher yield plasmid extraction, 100 ml of bacterial culture was subjected to Plasmid Midi Kit (QIAGEN). Cloned plasmids were sequenced using psiCheck2_hLucF and psiCheck2_hLucR for screening.

### Dual luciferase assay

Approximately 2 × 105 HEK293T cells were plated in each well of 12-well plate the day before transfection. Cells were transfected with either plasmids overexpressing miRNAs, LentimiRa-GFP-mmu-mir-146a/221/656 (mm10082/mh10296/mh10968, ABM Good) or empty vector LentimiRa-GFP-empty (m001, ABM Good), together with psiCheck2_GRIA3_UTR, psiCheck2_KCNK2_UTR, or psiCheck2_MAP2_UTR plasmids (ratio 1:3 to ensure good expression of luciferase). Transfection was performed using JetPRIME® Polyplus Transfection Reagent (Ozyme) following manufacturer’s instruction. Assay was performed using Dual-Luciferase® Reporter Assay System (Promega) 24 h after transfection. Ratio of Renilla luciferase to firefly luciferase was taken as mean of technical triplicates.

### In situ hybridization analysis

#### Tissue preparation and processing

Male, 2-month-old wild type mice were deeply anesthetized using pentobarbital and fixed by intracardiac perfusion with 4 % paraformaldehyde in phosphate-buffered saline (PBS). Brains were removed, post-fixed overnight at 4 °C, and cryo-protected using two baths of Tris-HCl buffered saline (TBS) with 0.5 M sucrose at 4 °C during 48 h. Brains were then frozen in bath of isopentane at −35–40 °C and stored at −80 °C. Serial sections of 20 μm were done in a cryomold (Tissue-Tek) with a cryostat (Leica), mounted on Superfrost Plus glass slides (Thermo Scientific), and preserved at −80 °C. For embryos and early postnatal animals, intracardiac perfusion was replaced by direct 4 % paraformaldehyde fixation overnight after decapitation.

#### In situ hybridization procedure

Tissue sections mounted on glass slides were thawed and air dried for 1 h at room temperature, incubated in a solution containing 20 μg/ml proteinase K (Sigma) in TBS, pH 7.4 for 3 min, and then washed for 5 min two times in TBS. Samples were fixed in 4 % paraformaldehyde (PFA) for 10 min and washed with 2 mg/ml glycine in TBS and twice for 5 min in TBS. To remove residual phosphate from the TBS washes, slides processed with 1-ethyl-3-(3-dimethylaminopropyl)carbodiimide (EDC) fixation were incubated twice for 10 min in freshly prepared solution containing 0.13 M 1-methylimidazole, 300 mM NaCl, pH 8.0 adjusted with HCL. In the meantime, a solution of 0.16 M EDC (Sigma) is prepared by adding EDC into 1-methylimidazole and 300 mM NaCl (pH 8.0) solution. The pH of the EDC solution is readjusted by adding 12 M HCl to pH 8.0. Slides are maintained in a humidified chamber for 1 h at room temperature incubated in 500 μl of EDC solution to each slide. The slides are then washed in 2 mg/ml glycine/TBS solution and then twice for 5 min in TBS. For enzymatic inactivation, slides were acetylated by incubating for 30 min in a solution of freshly prepared 0.1 M triethanolamine and 0.5 % (*v*/*v*) acetic anhydride. Slides were then rinsed twice for 5 min in TBS. For pre-hybridization, the tissue sections were covered with 500 μl of hybridization buffer containing 50 % formamide, 5× SSC, 5× Denhardt’s solution, 0.25 mg/ml yeast transfer ribonucleic acid (tRNA), 0.5 mg/ml salmon sperm DNA, 20 mg/ml blocking reagent (Roche), 1 mg/ml 3-((3-cholaminodopropyl) dimethylammonio)-1-propanesulfate (CHAPs, Sigma), and 0.5 % tween at room temperature for 2 h in a humidified chamber. The hybridization buffer was removed by tilting the slide. Sequences of the different probes are described in Additional file 1: Table S4. For hybridization, 40 nM of DIG-labeled locked nucleic acids (LNA) probe (mmu-miR-146a, mmu-miR-scramble) diluted in hybridization buffer were applied per section and covered with coverslips. The slides were incubated in a sealed humidified chamber for 16 h at 42 °C.

Slides were immersed for 3 min in 2× SSC at 52 °C and then washed for 5 min twice in 1× SSC at 52 °C and once in 1× SSC at room temperature. Slides were washed again for 5 min twice in 0.2× SSC at room temperature and once in TBS. In preparation for probe detection, 500 μl of blocking solution containing 5 mg/ml blocking reagent (Roche), 10 % (*v*/*v*) normal goat serum, and 0.1 % (*v*/*v*) tween in TBS was applied to each slide for 1 h at room temperature in a humidified chamber. Antibody anti-DIG-FAB peroxidase (POD) (Roche) diluted 1:500 in blocking solution was incubated overnight. Slides were then washed three times in TBS. Slides were incubated in freshly prepared NBT/BCIP substrate reagent containing 50 mM MgCl2, 100 mM NaCl, 0.2 mM Levamisole, 0.5 mg/ml NBT, and 0.1875 mg/ml BCIP in 100 mM TBS pH 9.5 and protected from light during the exposure time (approximately 6 h). Slides were then washed twice in water, then twice in TBS and incubated for 10 min in 4 % PFA solution. Finally, slides were washed in water and mounted using two drops of mounting medium.

### Immunohistochemistry and miRNA ISH co-staining

To assess cellular localization of miR-146a, brain sections were processed for both immunofluorescence staining and in situ hybridization (ISH) staining. Slides were washed three times in PBS and then incubated 1 h at room temperature in humidified chamber, with 500 μl of blocking solution containing 0.1 % (*v*/*v*) triton-100× and 2 % (*v*/*v*) normal donkey serum in PBS. The blocking solution was removed by tilting the slide. Slides were incubated overnight at room temperature with the same blocking solution containing polyclonal antibody against Fox3/NeuN (1:1000; Abcam, AB104225) for specific neuronal staining and antibody against GFAP (1:500; Abcam, ab7260). Slides were rinsed three times in PBS and incubated for 1 h 30 min at room temperature with donkey Alexa fluor 488-conjugated anti-rabbit (for NeuN), secondary antibody diluted in blocking solution. After being rinsed in PBS, then water, slides were mounted in mounting medium.

### Microscopy and image processing

Images were captured on an upright epifluorescence microscope, Nikon Eclipse Ni-U (Nikon France S.A) using 10× objective CFI Plan Fluor NA 0.30 and 40× objective CFI Plan Fluor NA 0.75. For fluorescent imaging filters, sets for GFP were used, and pictures were acquired using a Zyla SCMOS camera (Andor Technology Ltd., Belfast, UK).

### Primary neuronal cultures

Cultured mouse hippocampal neurons were prepared from E16.5 embryos, grown on 18-mm glass coverslips coated with laminin/polylysine and maintained in Neurobasal B27-supplemented medium. Neurons were transfected using Lipofectamine 2000 on days in vitro 11 (DIV11) with 800 ng of plenti-III-miR-GFP-Blank or pLenti-III-miR146a-GFP, and experiments were performed at DIV18.

### Sholl analysis

Analysis of the dendritic morphology was done blind to the genotype. Sholl analysis was done using a Sholl analysis pluggin on ImageJ and determined the number of dendritic branch intersections with concentric circles of increasing radii (interval of 10 μm) from the soma. All neurons were imaged at the same magnification, and images were threshold, and to avoid false intersections because of noise, each threshold images were checked and compare to the original. The BI (branching index) was shown: The BI compares the difference in the number of intersections made in pairs of circles relative to the distance from the neuronal soma, following the following equation: BI = ∑(intersections circle *n* − Intersections circle *n* − 1).rn according to “A new mathematical function to evaluate neuronal morphology using the Sholl analysis: Luis Miguel Garcia-Segura, Julio Perez-Marquez”.

### Virus production

Lentivirus was produced using lentiviral compatibility vectors as mentioned above by the Plateforme Vecterus Viraux et Transfert de Gènes from Hospital Necker as paid service. Viral titre was determined by FACS of GFP signal. For all experiments, virus infection was performed at the multiplicity of infection (MOI) of 2 in normal media; virus media was replaced after at least 4 h of incubation.

### Primary astrocyte culture

Astrocytes were extracted from cortexes of P1 mice and grown on uncoated flask in DMEM Low Glucose Medium GlutaMAX™ (Life Technologies) supplemented with Penicillin-Streptomycin (100 U/ml) (Life Technologies) and 10 % FBS. When cell reach 70–80 % confluent, they were detached with 0.05 % Trypsin-EDTA (1×) Phenol Red (Life Technologies) and replated in desired cell density; this passaging step was only performed once for each culture. For cell growth assay, 20,000 cells were replated into E-Plate 96 for continuous monitoring on the Xcelligence RTCA-MP instrument (ACEA Biosciences) for 5 h before being infected with virus; growth rate was measured over 72 h during which cell index was analyzed every 15 min. Growth rate was measured as slope of normalized cell index from at least three technical triplicates over a set period of time; this analysis was performed using RTCA Software V2.0 (ACEA Biosciences).

For glutamate uptake assay, 200,000 cells were replated into a 24-well plate and allowed to reach 100 % confluent. Cells were then treated with a mitotic blocker 8 μM cytosine β-d-arabinofuranoside (Sigma) for 5–6 days followed by a 90-min incubation with 75 mM l-leucine methyl ester (Sigma) to completely diminish the culture of microglia; viral infection was performed overnight. The next day, cells were serum starved for 2 h before being incubated in media containing 1 μCi/ml (H3)-glutamic acid (Perkin Elmer) and 50 μM of unlabeled glutamate (Sigma); uptake was allowed in the incubator for 30 min before cells were washed three times with ice cold Hanks’ balanced salt solution (Life Technologies) supplemented with HEPES 15 mM (Life Technologies) and lysed in 1 M NaOH 0.1 % Triton-X solution at 42 °C for 10 min. For negative control, the transporters were blocked using 250 μM L-trans-pyrrolidine-2,4-dicarboxylic acid (PDC) (Sigma) (maintained even during the serum starved step). Radioactivity was measured as average count per minute during 10 min. Rate of uptake was measured from duplicates, normalized to samples treated with PDC, and the protein concentration was measured by Bradford assay.

## Results

### Identification of miRNA deregulation signature in ASD

**Fig. 1:**
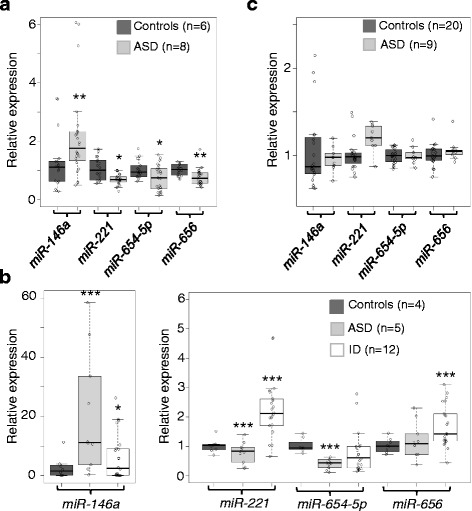
Identification of a conserved miRNA signature in ASD. **a** Significantly deregulated miRNAs after validation in ASD OMSC after the validation round, representing the miRNA deregulation signature of ASD: *miR-146a*, *miR-221*, *miR-654-5p*, and *miR-656* (±SD, controls—*dark gray bar*, patients—*light gray bar*). *Dots* represent average of technical triplicates for each biological repeat. *P < 0.05, **P < 0.01 by Wilcoxon rank sum test. **b** Tissue and disease specificity of miRNA signature. Expression (±SD) of *miR-146a*, *miR-221*, *miR-654-5p*, and *miR-656* were assessed in primary skin fibroblasts of ASD patients (*n* = 5, *light gray bar*), ID patients (*n* = 12, *white bar*), and controls (*n* = 4, *dark gray bar*). Results were obtained from Taqman assays performed on Fluidigm array. *Dots* represent average of technical triplicates for each biological repeat. *P < 0.05, ***P < 0,001 by Student’s paired two-tailed *t* test. **c** Expression (±SD) of miRNA signature in peripheral blood mononuclear cells (PBMC). No significant difference in miR expression was detected between the controls (*n* = 20, *dark gray box*) and the ASD patients (*n* = 9, *light gray box*). Results were obtained from Taqman assays performed on Fluidigm array. *Dots* represent average of technical triplicates for each biological repeat

We harvested OMSCs from the lamina-propria layer of the nasal cavities of living ASD patients and age and sex matched controls (see Additional file 1: Table S1) and profiled 667 miRNAs (Sanger miRBase v10) in eight patients and six controls using the TLDA high-throughput real-time qPCR approach. The genetic etiology of these patients is mostly unknown according to whole exome sequencing and array comparative genomic hybridization analyses (see Additional file 1: Tables S2, S3). In keeping with the current gold standard in miRNA analysis, miRNA relative quantity were calculated using ΔCt method using the geometrix mean of seven house-keeping miRNAs *miR-let-7g*, *miR-106a*, *miR-151-3p*, *miR-15b*, *miR-16*, *miR-17*, and *miR-99b* (see Additional file 1: Figure S1) and the average of all controls for normalization [21, 22]. Twenty four miRNAs which were either found significantly deregulated in the patient groups (corrected *P* values <0.05) or for which the PCR assay failed originally were included in the second round for validation (see Additional file 1: Figure S1). These analyses identified four miRNAs, *miR-146a* (upregulated twofold), *miR-221*, *miR-654-5p*, and *miR-656* (all three down-regulated ~1.3-fold), significantly deregulated in the patients (*P* < 0.05) (see Fig. 1a). *miR-654-5p* and *miR-656* are conserved only in the primate chain and are expressed at low level in the human brain; by contrast, *miR-146a* and *miR-221* sequences are 100 % conserved in the mouse and are highly expressed in different human brain regions throughout development including the cortex, hippocampus, and cerebellum (Allen Brain Atlas, Brain Span) [23], supporting a role in development.

### Tissue and disease specificity of the miRNA signature

We next questioned the tissue specificity of this miRNA signature and tested miRNA expression in different cell types. For this, we collected either primary skin fibroblasts from 5 new ASD patients and 4 controls or peripheral blood mononuclear cells (PBMC) from 9 ASD patients (3 of whose fibroblasts were also analyzed) and 20 controls (see Additional file 1: Table S5). Indeed, primary human fibroblasts are readily accessible untransformed cells that have been previously used as model for neuropsychiatric disorder studies [24, 25], and PBMCs are suitable for screening of large cohorts. Since no prior standard for reference miRNAs in fibroblast or PBMC exist, we analyzed the same 7 reference miRNAs (*miR-let-7g*, *miR-106a*, *miR-151-3p*, *miR-15b*, *miR-16*, *miR-17*, and *miR-99b*) as the above analysis and U6, a non-coding RNA normally used as reference for miRNA assay. We excluded miRNAs with the most variations between groups, *miR-99* and *U6* (in fibroblasts) and *miR-16* (in PBMC), from being used as reference miRNAs (see Additional file 1: Figure S2, S3). This analysis showed a significant deregulation of *miR-146a*, *miR-221*, and *miR-654-5p* in the ASD group compared to controls in the same trend as in OMSC (see Fig. 1b). By contrast, no differential expression of the four miRNAs could be detected in PBMC from ASD patients compared to controls (see Fig. 1c). We then raised the issue of the disease specificity of this signature in related neurodevelopmental disorders and extended the analysis to primary skin fibroblasts from patients who have known causes of intellectual disability (ID) without autistic features (*n* = 12) (see Additional file 1: Table S6). Our results showed that ID patients do not share the same signature as ASD (see Fig. 1b). Yet, *miR-146a* was also found significantly upregulated in the ID group, supporting the role of this miRNAs in shared pathways affected in both conditions. Moreover, the level of *miR-221*, *miR-656*, and *miR- 654-5p* transcripts observed in the ID group suggests that these miRNAs could be used to develop a diagnostic panel allowing the distinction between the ASD, ID, and the control groups.

### miRNA expression deregulation is not mediated by DNA variations

To get insight into the mechanism underlying deregulated miRNA expression, we search for DNA variation in the miRNA regions that could account for the differential expression observed between ASD and controls. We first re-analyzed the results of the array comparative genomic hybridization and whole exome sequencing analyses previously performed for all patients. Neither copy number nor single nucleotide polymorphism (SNP) was identified in the exonic region nor in the immediate captured region flanking the miRNAs. The presence for variant within the promoter region could not be investigated as the corresponding sequences have not been precisely defined. Yet, we performed capillary sequencing to assess the distribution of a known expression quantitative trait locus SNP (*rs57095329*) in the promoter of *miR-146a* in both cohorts (control and patient) [26]; only one patient fibroblast sample carries this SNP, ruling out its contribution to the expression difference detected. Collectively, these data suggest that the abnormal miRNA expression observed in ASD patients’ cells is likely due to transcriptional deregulation or altered posttranscriptional processing of pri-miR transcripts.

### ASD-deregulated miRNAs target neuronal relevant genes and pathways

**Fig. 2:**
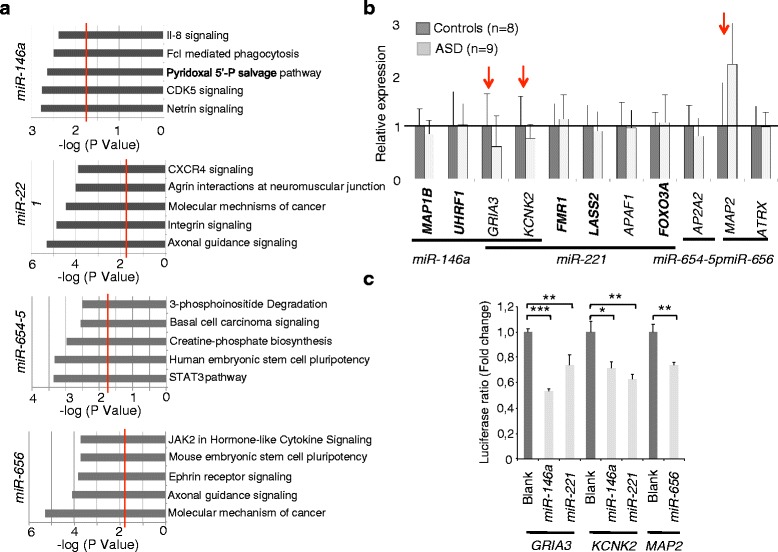
miRNAs directly regulate neuronal relevant genes. **a** Gene ontology enrichment of predicted miRNA targets by ingenuity pathway analysis (IPA). About 1000 genes were predicted to be regulated by each miRNA by at least three different prediction programs and were included in the analyses. Only the top 5 enriched pathways are shown. *P* values were calculated by Fisher’s exact test; *red line* represents correction threshold by Bonferroni Correction. **b** Mean expression (±SD) of known (*in bold*) and predicted targets of miRNAs in ASD (*n* = 9, *light gray bar*) and control OMSCs (*n* = 8, *dark gray bar*). Gene expression was measured using relative standard curve method, normalized against *GPBP1* as reference gene. Results shown represent one of two independent repeats showing the same results. **c** Western blot showing down regulation of KCNK2 in ASD (*n* = 3) with respect to control OMSCs (*n* = 3). ACTB was used as loading control. *Bottom panel* displays densitometry of the bands using two images taken at low exposures. *P < 0.05 by Student’s paired two-tailed *t* test. **d** The 3′UTRs of *GRIA3*, *KCNK2*, and *MAP2* are targeted by miRNAs. The 3′UTRs of *GRIA3*, *KCNK2*, and *MAP2* were subcloned into the 3′UTR of Renilla luciferase in the psiCheck2 plasmid and co-transfected into HEK293T with either plasmid overexpressing *miR-146a*, *miR-221*, and *miR-656* or empty plasmid. Ratio of Renilla/firefly luciferase (±SD) indicates the repression activity of miRNAs directly on the 3′UTR. Results are represented from one repeat of two showing the same results. *P < 0.05, **P < 0.01, ***P < 0.001 by Student’s paired two-tailed *t* test

Next, we searched for putative messenger RNA (mRNA) targets deregulated in patients’ OMSCs. Using miR-DIP to integrate target prediction from multiple programs, we found that on average, 1000 targets predicted for each miRNA by at least three different prediction programs. We integrated the prediction analyses with that of pathway enrichment using ingenuity pathway analysis and identified several highly relevant pathways (see Fig. 2a and Additional file 1: Table S7). These are neuronal pathways, including axonal guidance signalling, signalling by Rho Family GTPases, actin cytoskeleton signalling, and synaptic long-term potentiation (see Additional file 1: Figure S4), as well as immunological pathways which include IL-8 signalling, CXCR4 signalling, and macropinocytosis signalling (see Fig. 2a).

To validate some of these in silico predictions, we assayed by RT-qPCR the transcript levels for several known and predicted miRNA targets involved in the identified pathways (see Fig. 2b). Albeit not significant, decreased levels of *GRIA3* and *KCNK2* transcripts and increased levels of *MAP2* mRNA were found in patients’ OMSCs (see Fig. 2b). Using a dual luciferase reporter assay, we confirmed that these transcripts are direct targets of the identified miRNAs. Overexpressing either *miR-146a*, *miR-221*, or *miR-656* significantly reduced the luciferase activity of reporter constructs carrying the 3′-untranslated region (3′-UTR) of *GRIA3*, *KCNK2*. and *MAP2* (see Fig. 2c).

### *miR-146a* and *miR-221* displays strong expression in regions important for high cognitive functions

**Fig. 3:**
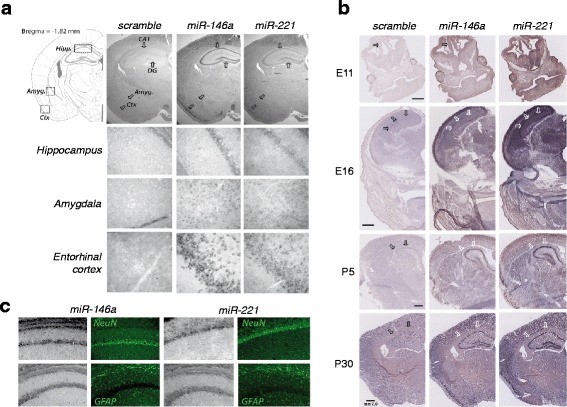
Expression of *miR-146a* and *miR-221* in the brain. **a** ISH of *miR-146a* and *miR-221* in the adult mouse brain. Typical coronal slices showed elevated *miR-146a* in the hippocampal CA1 and dentate gyrus (*DG*), the amygdala nuclei (*amyg*), and the entorhinal cortex (*ctx*). **b** Hybridization of scramble or *miR-146a* and *miR-221* was performed onto mice at different developmental from embryonic (E11 and E16) and postnatal (P5 and P30) stages. *Arrows* indicate hippocampal formation or territories at the different age, allowing appreciation of the specific labeling of different cellular layers in the postnatal brains. **c** Neuronal expression of both miRNAs was confirmed by combining miR-ISH with fluorescent labeling of neuronal (NeuN) and glial (GFAP) cells in the CA1 region

In the mouse brain, the expression pattern of *miR-146a* and *miR-221* strongly correlates with that in the human brain (Allen Brain Atlas, Brain Span); they are expressed throughout the cortex, hippocampus, and amygdala as evidenced by in situ hybridization (see Fig. 3a). During development, both miRNAs exhibit an initially high and widespread expression that becomes restricted to some cellular layers in the above-cited postnatal brain regions (see Fig. 3b). In situ hybridization associated with the immune-detection of cell-specific markers showed that in the adult mouse brain, *miR-146a* and *miR-221* are essentially expressed in neurons (see Fig. 3c), whereas very few signal was detected in the glial lineage (see Fig. 3c).

### *miR-146a* overexpression affects astrocyte glutamate uptake capacity and neuronal dendritic arborization

**Fig. 4:**
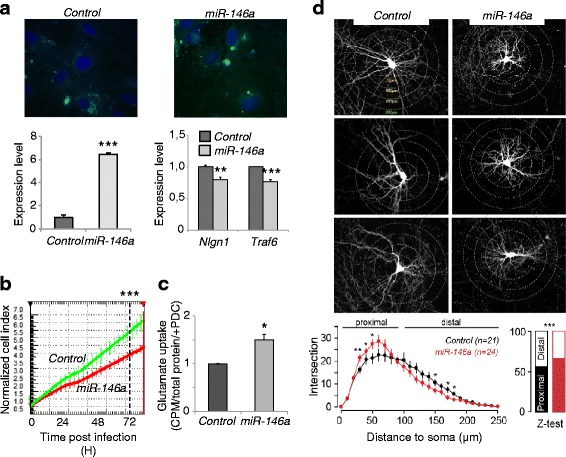
*miR-146a* overexpression alter neuronal and glial cells biology. **a** Efficacy of viral induction 24 h post-infection with lentivirus carrying the empty vector (control) or *miR-146a. Top panel* shows primary mouse astrocyte expressing GFP; DAPI was used to counterstain the nucleus. *Bottom left panel* shows *miR-146a* level (±SD) induced by the virus; expression was measured by Taqman assay and normalized against *miR-221* in technical triplicates; *bottom right panel* shows the expression of *Nlng1* and *Traf6* (±SD) measured by RT-qPCR in technical triplicates, normalized against *Actb*; **P < 0.01, ***P value <0.001 by Student’s paired two-tailed *t* test. **b** Growth of primary astrocyte is significantly impaired by *miR-146a* upregulation (*red line*) compared to the control (*green line*). Cell growth, displayed as normalized cell index (±SD), was continuously monitored using the Xcelligence system over 4 days. Statistic analysis was performed using data collected from technical quadruplicates at 72 h post-infection. The *graph* is from one repeat of three showing the same results. ***P value <0.001 by Student’s paired two-tailed *t* test. **c** Glutamate uptake is significantly increased in astrocytes overexpressing *miR-146a*. Following a published protocol [53], uptake of radioactive glutamate (±SD) was counted as count per minute (CPM), normalized against total protein and CPM values of samples treated with the glutamate transporter blocker PDC. The *graph* is from one repeat of three showing the same results. *P value <0.05 by Student’s paired two-tailed *t* test. **d** *miR-146a* upregulation inhibits neurite outgrowth. Sholl analysis of dendritic arborisation of control and *miR-146a*-overexpressing neurons. *P < 0.05; **P < 0.01. To test for differences in intersections between blank and *miR-146a* neurons, each distance (10 μm steps) was tested by one-way ANOVA statistical test followed by the Holm-Sidak post hoc test. When data where not following a normal distribution, we used the one-way ANOVA on ranks and Dunn’s method for post hoc test

The current consensus is that ASD physiopathology is based on either defective neural connectivity, excitatory-inhibitory neural activity imbalance, disturbed dendritic morphology, or neuroimmune disturbances. Therefore, we tested whether *miR-146a* overexpression could affect any of these processes using primary neuronal and glial cell cultures transduced with lentiviral constructs driving *miR-146a* expression. Primary astrocytes were cultured from cortexes of P1 mice and infected with lentiviral virus at multiplicity of infection of 2 (efficacy 100 %) to induce roughly 6.5-fold upregulation of *miR-146a* (see Fig. 4a). Significant reduction of known targets, *Nlgn1* [27] and *Traf6* [28], was confirmed by RT-qPCR (see Fig. 4a). Compared to astrocytes infected with the empty lentiviral vector (control), astrocytes overexpressing *miR-146a* displayed a significant decrease in growth rate as assessed by Xcelligence growth assay (see Fig. 4b), consistent with previous published evidence [29]. Most interestingly, these astrocytes also exhibited a significant increase in glutamate uptake capacities (see Fig. 4c), suggesting a defect in either the regulation of the transport and/or processing and/or the metabolism of the glutamate. Quantification of the transcript and protein levels of the two glial specific glutamate transporters (SLC1A2 and SLC1A3), the glutamate dehydrogenase (GLUD1), and the glutamine synthetase (GLUL) failed to identify any marked alteration (data not shown). The precise mechanism of how *miR-146a* controls this core function of astrocyte remains to be investigated.

To further explore the consequence of *miR-146a* upregulation on neuronal cells morphology, we assessed dendritic arborization extent and complexity of primary neuronal cultures transduced with the same lentiviral constructs using Sholl analysis. When compared to GFP expressing cells transfected with a blank construct, *miR-146a* overexpressing neurons displayed shriveled dendritic trees with branching points occurring more proximal than in control conditions (see Fig. 4d). This suggests that the level *miR-146a* expression in also an important determinant for neuronal development.

## Discussion

We report here a comprehensive analysis of miRNA expression profiles in human stem cells from ASD patients. We identified a signature of four miRNAs (*miR-146a*, *miR-221*, *miR-654-5p*, and *miR*-*656*) commonly deregulated in ASD. This signature is not conserved in PBMCs, a result consistent with the two previously reported miRNA expression studies performed in serum from children with autism [30, 31], but is also observed in primary skin fibroblasts from unrelated patients. While this observation may restrict the ease to rapidly validate our data in a larger cohort, it highlights the relevance of OMSCs, a neurologically relevant tissue, for understanding the pathophysiological mechanisms underlying neurodevelopmental disorders and supports the use of primary fibroblasts for routine testing. Notably, comparable miRNA profiling in OMSCs was recently performed in the context of schizophrenia [32], and similar results were obtained with increased *miR-382* expression observed in OMSCs samples from patients but not in peripheral blood-derived/non-neuronal samples. Despite the limited number of samples tested, potentially restricting the statistical power, the observed conservation of the signature in primary skin fibroblasts from unrelated patients strongly supports its biological relevance. Lastly, our finding that ID patients do not share the same signature as ASD may have diagnostic implications and raises the possibility of using miRNA signature as a biomarker for ASD, providing a means for detecting early signs of the disease and allowing early intervention.

Target prediction analysis suggests that the transcripts regulated by these miRNAs code for proteins involved in neurodevelopmental processes as well as immune response and inflammation, both of which are relevant for the pathology of ASD. Previous studies have demonstrated that immune imbalance impairs higher order brain functioning and altered inflammatory responses have been reported in ASD patients [33, 34]. Moreover, DNA methylation analysis of the autistic brains has identified a very significant enrichment of differentially methylated region in genomic areas responsible for immune functions [5]. Along these lines, it is noteworthy that *miR-146a* was first identified as an immune system regulator and possibly plays an important role in neuroinflammation [28]. Our results support the hypothesis that altered *miR146a* expression contributes to the neuroinflammatory processes reported in the autistic brain.

*miR-146a* and *miR*-*221* have also established roles in neurodevelopment. Validated targets of *miR-146a* include *MAP1B*, which regulates AMPA receptor endocytosis [35], while direct targets of *miR*-*221* include *FMR1*, the fragile X gene which is the most frequent cause of syndromic intellectual disability (ID) and ASD [36]. Using target prediction tools combined to dual luciferase assays and Western Blot analysis, we identified additional neuronal transcripts regulated by these miRNAs. We showed that *GRIA3* is a target transcript for both *miR-146a* and *miR-221. GRIA3* encodes a core subunit of the AMPA receptor, and findings from various experimental systems implicate ionotropic GluR dysfunction in ASD [37]. *miR-146a* upregulation in ASD neurons might thus alter AMPA receptor biology through both impaired MAP1B-mediated endocytosis and decreased amount of *GRIA3*. We also demonstrated that miRNA expression deregulation correlates with a reduced level of KCNK2 protein in patients’ OMSCs and that both *miR-146a* and *miR-221* directly target *KCNK2* 3′UTR. *KCNK2* is a member of the potassium leak channel family, a group of proteins that are critical determinants of neuronal excitability in the cortex [38], and *KCNK2* knockdown impairs neuronal migration in the developing mouse cerebral cortex [39]. Reduced amount of KCNK2 protein may thus contribute to the failure or delay in neuronal migration that has been observed in some cases of autism and ID.

One prevalent theory for the pathogenesis of autism relates to a deficiency in the plasticity of axonal sprouting and synaptic connectivity. There have been reports in autism cases of enlarged or abnormally oriented neurons, densely packed neuronal regions, and isolated regions of atrophic neurons with reduced dendritic arborisation [40, 41]. Increased spine density has also been reported in fragile X syndrome [42]. Conversely, neuronal atrophy and reduced dendritic spines is mentioned in Rett syndrome [43]. Our observation that *miR-146a* overexpression disturbs neuronal dendritic arborization is thus consistent with the defective neural connectivity found in ASD.

*miR-146a* has also been linked to the processes underlying glial cells differentiation, and previous reports demonstrated that it inhibits the expression of neuron-specific targets *Nlgn1* and *Syt1*, preventing glial cells from mistakenly adopting neuron-specific phenotypes [27]. Using in vitro assays, we demonstrated that *miR-146a* overexpression alters astrocyte glutamate uptake capacity. Interestingly, these findings coincides with our growing knowledge for the role of glial cells in every aspects of the development and function of neuronal cells, with the recent evidence that astrocytes play a major role in neurodevelopmental disorders and the demonstration of altered glutamatergic synaptic transmission in ASD [44, 45]. We propose that abnormal *miR-146a* expression impairs astrocyte differentiation and function in ASD brain and that altered synaptic clearance of glutamate may contribute to the excitatory-inhibitory activity imbalance underlying autism.

Remarkably, *miR-146a* overexpression is not restricted to ASD patients. First, we demonstrated that *miR-146a* overexpression is also observed in cells from patients with ID. Second, increased amount of *miR-146a* has been found in epilepsy-associated glioneuronal lesions [46], in serum of epilepsy patients [47] as well as in the hippocampus in a rat model of temporal lobe epilepsy [48]. *miR-146a* upregulation seems thus to be a common hallmark of different neurodevelopmental disorders and may mediate common pathways underlying these conditions. Understanding how this miRNA contributes to the interactions between synaptic function and immune molecules and cells will therefore have broad implications.

Lastly, our findings may have important consequences for the development of future therapies, as several strategies have been established to restore miRNA levels or block miRNA function. The expression levels of particular miRNAs can be restored either with miRNA mimics or with miRNAs encoded in expression vectors. Conversely, current strategies for inhibitory targeting of microRNAs are mainly based on antisense oligonucleotides (so-called anti-miRNAs), comprised of locked nucleic acids (LNA) along with tiny LNA anti-miRNA constructs, antagomirs, and miRNA sponges [49–51]. Indeed, it was recently shown that intracerebroventricular injections of *antagomir-155* and *antagomir-802* in the Ts65Dn mice, a Down syndrome mouse model that overexpress such miRNAs, resulted in the attenuation of 30–40 % of endogenous *miRNA-155* and *miR-802* and increased *Mecp2* target gene expression 7 days after administration [52]. While manipulating miRNAs expression to achieve biological levels still requires optimization, our results support the idea that it might be a promising strategy in the field of ASD. Further studies are needed to address the precise molecular mechanisms underlying miRNA expression deregulation, and whether this is the consequence of a transcriptional defect or of a miRNA maturation processing defect need further clarification. Investigation in animal models will also be necessary to determine the exact impact of *miR-146a* and *miR-221* deregulation on synaptogenesis and synaptic plasticity.

## Conclusions

We identified a signature of four miRNAs commonly deregulated in ASD and show that this signature is partially conserved in primary skin fibroblasts. While the final demonstration of our findings is awaiting the replication across a larger number of patients, we propose that this miRNA signature may allow discriminating between ASD and intellectual disability samples.

The deregulated miRNAs target neuronal relevant genes and pathways, and we demonstrated altered levels of corresponding transcripts in patients’ cells. More importantly, we demonstrated that abnormal expression of one of these miRNAs (*miR-146a*) alters neuronal dendritic complexity and astrocyte glutamate uptake capacities, providing a convincing link between miRNAs deregulation and ASD pathophysiology.

## Additional files

Additional file 1:
**Profiling olfactory stem cells from living patients identifies miRNAs relevant for Autism pathophysiology.** **Figure S1.** Relative expression of miRNAs in OMSC in validation round.** Figure S2.** Expression of reference miRNAs in fibroblasts. **Figure S3.** Expression of miRNA signature in PBMC. **Figure S4.** Long term potentiation pathway is predicted to be specifically targeted by *miR-146a and miR-221*. **Table S1.** Clinical descriptions of ASD patients whose OMSCs were used for miRNA screening. **Table S2.** Variants identified by whole exome analysis. **Table S3.** Genetic etiology of patients included in this analysis. **Table S4.** Primers used in this study. **Table S5.** Brief clinical descriptions of ASD patients whose primary skin fibroblasts and/or PBMC were used in follow up study. **Table S6.** Brief clinical descriptions of patients with ID whose primary skin fibroblasts were used in follow up study. **Table S7.** Selected enriched pathways predicted to be targeted by *miR-146a* and *miR-221*. (DOCX 1821 kb) Additional file 2:
**Raw expression value.** (XLSX 115 kb)

## Abbreviations

ASD: autism spectrum disorders
ID: intellectual disability
ISH: in situ hybridization
LNA: locked nucleic acids
miRNA: microRNA
OMSCs: olfactory mucosal stem cells
PBMC: peripheral blood mononuclear cells
SNP: single nucleotide polymorphism
UTR: untranslated region
