# Supplementary material for: Profiling olfactory stem cells from living patients identifies miRNAs relevant for autism pathophysiology
Source: Mol Autism. 2016 Jan 8;7:1. doi: 10.1186/s13229-015-0064-6 (PMC4705753; doi:10.1186/s13229-015-0064-6)
Supplement: Additional file 1: — Profiling olfactory stem cells from living patients identifies miRNAs relevant for Autism pathophysiology. Figure S1. Relative expression of miRNAs in OMSC in validation round. Figure S2. Expression of reference miRNAs in fibroblasts. Figure S3. Expression of miRNA signature in PBMC. Figure S4. Long term potentiation pathway is predicted to be specifically targeted by miR-146a and miR-221. Table S1. Clinical descriptions of ASD patients whose OMSCs were used for miRNA screening. Table S2. Variants identified by whole exome analysis. Table S3. Genetic etiology of patients included in this analysis. Table S4. Primers used in this study. Table S5. Brief clinical descriptions of ASD patients whose primary skin fibroblasts and/or PBMC were used in follow up study. Table S6. Brief clinical descriptions of patients with ID whose primary skin fibroblasts were used in follow up study. Table S7. Selected enriched pathways predicted to be targeted by miR-146a and miR-221. (DOCX 1821 kb) [file 13229_2015_64_MOESM1_ESM.docx]

***Identification of a common set of microRNAs deregulated in Autism Spectrum disorders***

Lam Son Nguyen, Marylin Lepleux, Mélanie Makhlouf, Christelle Martin, Julien Fregeac, Anne Philippe, François Feron, Bruno Gepner, Claire Rougeulle, Yann Humeau & Laurence Colleaux

This document contains 4 figures and 7 tables :

**Figure S1 :** Relative expression of miRNAs in OMSC in validation round.

**Figure S2 :** Expression of reference miRNAs in fibroblasts

**Figure S3 :** Expression of miRNA signature in PBMC

**Figure S4 :** Long term potentiation pathway is predicted to be specifically targeted by *miR-146a* and *miR-221*.

**Table S1 :** Clinical descriptions of ASD patients whose OMSCs were used for miRNA screening.

**Table S2 :** Variants identified by whole exome analysis**.**

**Table S3 :** Genetic etiology of patients included in this analysis.

**Table S4 :** Primers used in this study.

**Table S5 :** Brief clinical descriptions of ASD patients whose primary skin fibroblasts and/or PBMC were used in follow up study.

**Table S6 :** Brief clinical descriptions of patients with ID whose primary skin fibroblasts were used in follow up study.

**Table S7 :** Selected enriched pathways predicted to be targeted by *miR-146a* and *miR-221*


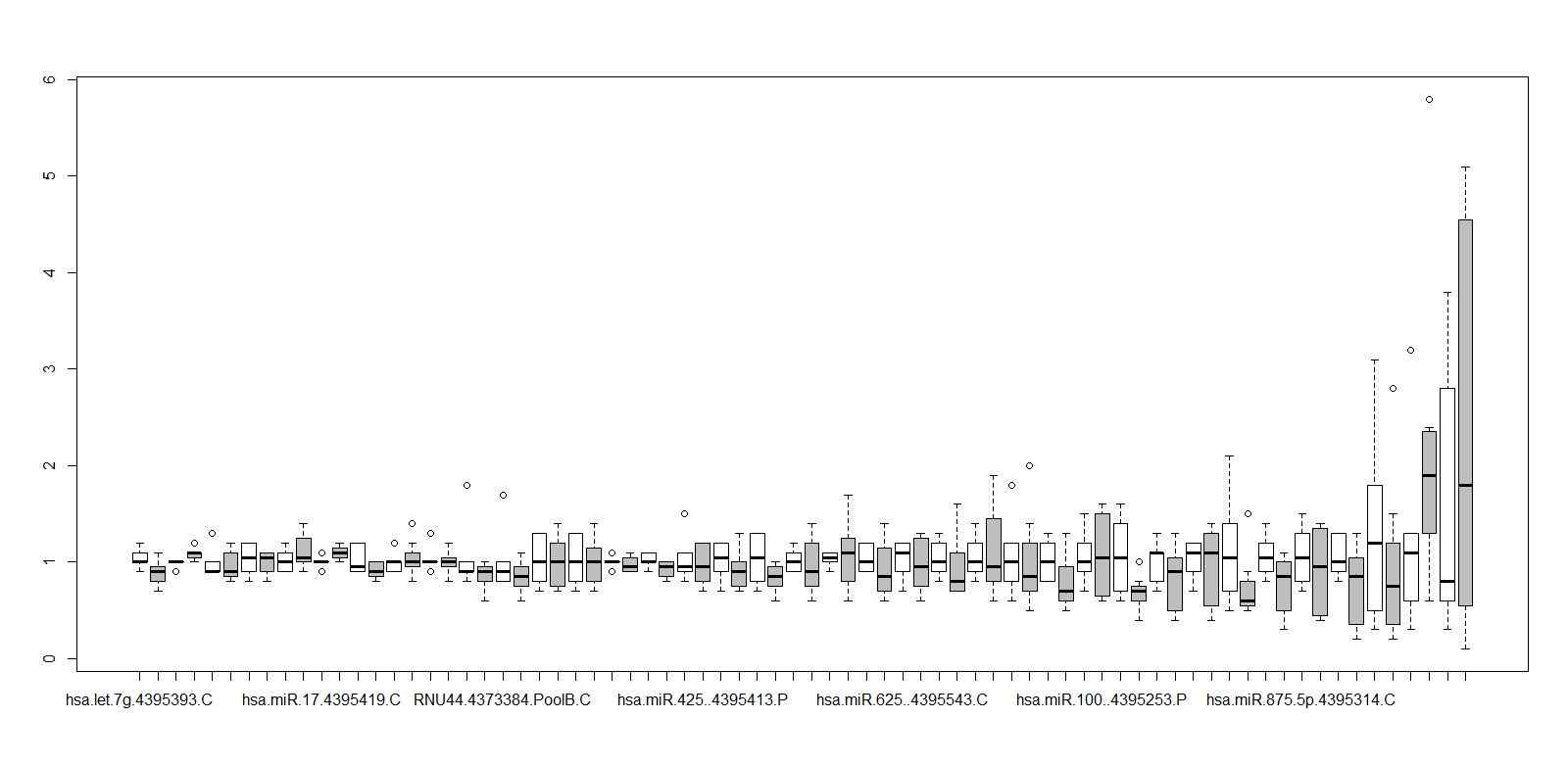


*hsa-miR-708*

*hsa-miR-146a*

*hsa-miR-335*

*hsa-miR-659*

*hsa-miR-432*

*hsa-miR-875*

*hsa-miR-100*

*hsa-miR-106b*

*hsa-miR-654-5p*

*hsa-miR-7-1**

*hsa-miR-513a*

*hsa-miR-222*

*hsa-miR-135a**

*hsa-miR-625**

*hsa-miR-505**

*hsa-miR-376a**

*hsa-miR-378*

*hsa-miR-656*

*hsa-miR-596*

*hsa-miR-221*

*hsa-miR-148b**

*hsa-miR-425**

*hsa-miR-126**

*hsa-miR-26b**

*

*

*

*


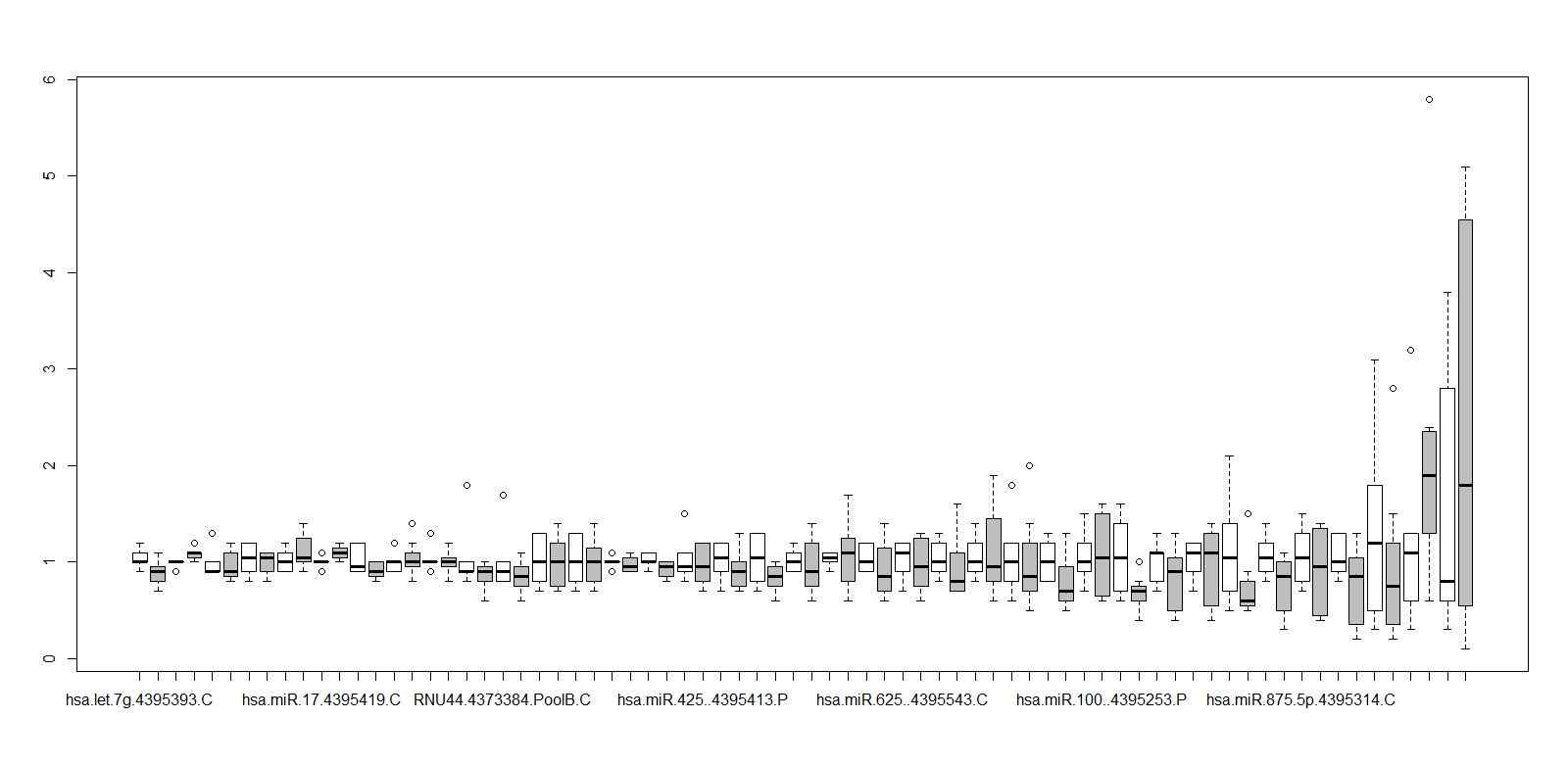


Controls (n=6)

Patients (n=8)

*hsa-let-7g*

*hsa-miR-106a*

*hsa-miR-151-3p*

*hsa-miR-15b*

*hsa-miR-16*

*hsa-miR-17*

*hsa-miR-99b*

Relative Quantity

Reference miRNAs

**Figure S1** : Expression (±SD) of all miRNAs in OMSC analysed in the validation round. * P < 0.05 by Wilcoxon rank sum test


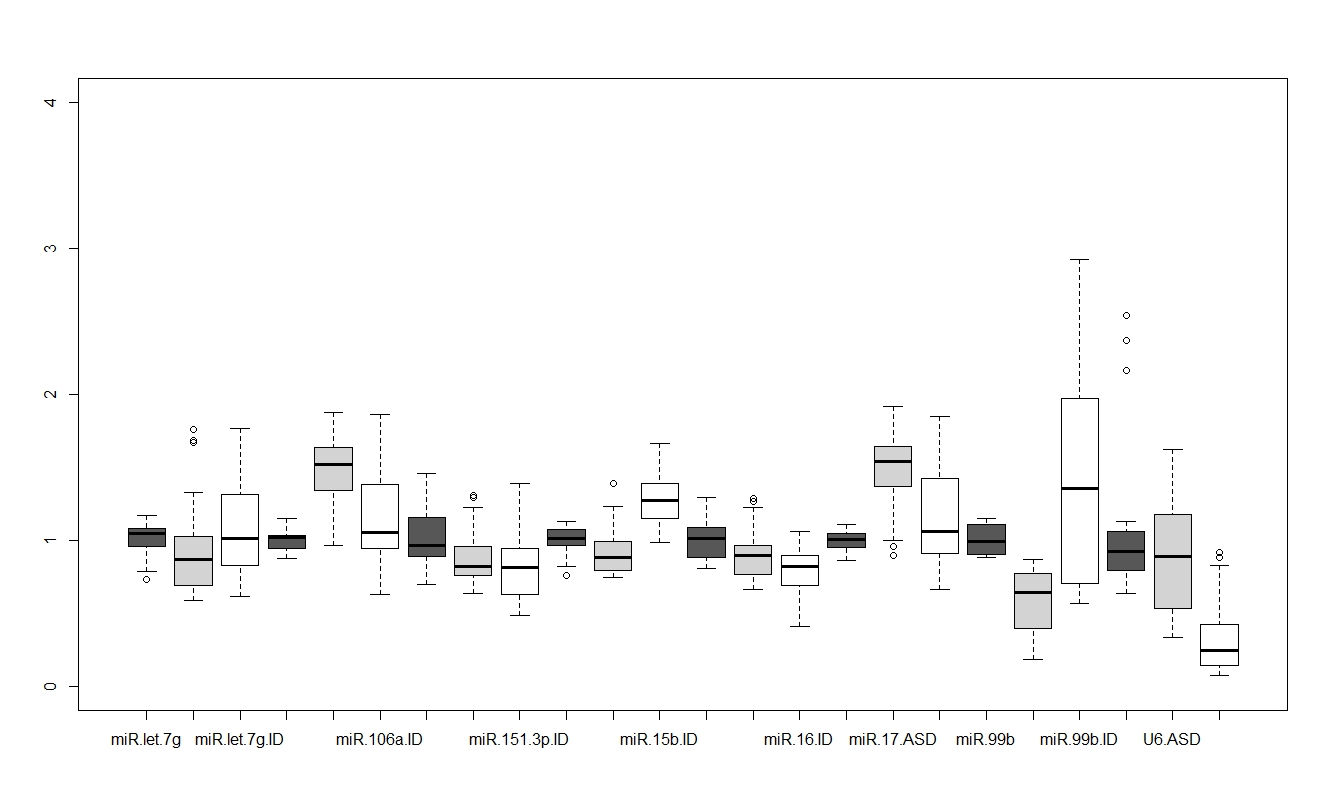


4

3

2

1

0

Controls (n=4)

ASD (n=5)

ID (n=12)

*miR-106a*

*miR-151-3p*

*miR-15b*

*miR-let-7g*

*miR-17*

*miR-99b*

*U6*

*miR-16*

Relative Quantity

**Figure S2** : Expression (±SD) of reference miRs used in Fludigm analyses in fibroblasts. 8 miRNAs were assessed including 7 identified and used in OMSC analysis and *U6*, the common housekeeping non-coding RNA for miR analysis. miRNAs with the most variation among groups were excluded from downstream analyses : *miR-99* and *U6*.

*miR-106a*

*miR-151-3p*

*miR-15b*

*miR-let-7g*

*miR-17*

*miR-99b*

*miR-16*

*U6*

3

2

1

0

4


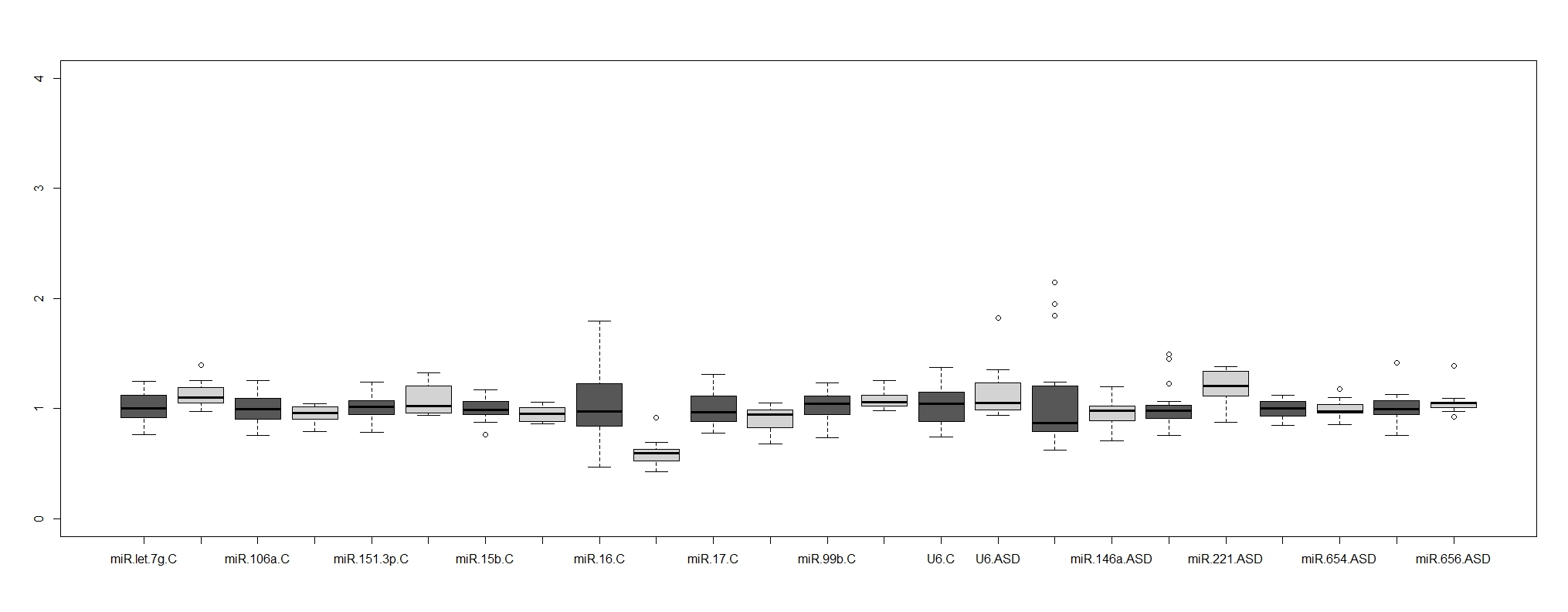


Controls (n=20)

ASD (n=9)

*miR-146a*

*miR-221*

*miR-654-5p*

*miR-656*

Relative Quantity

**Figure S3** : Expression (±SD) of miRNA signature in peripheral blood mononuclear cells (PBMC). *miR-16* was excluded from the final reference miRNA panel which include 6 miRNAs originally used OMSC analysis and *U6*, the common reference non-coding miRNA. No significant difference was detected between the controls (n=20, dark grey box) and the ASD patients (n=9, light grey box).


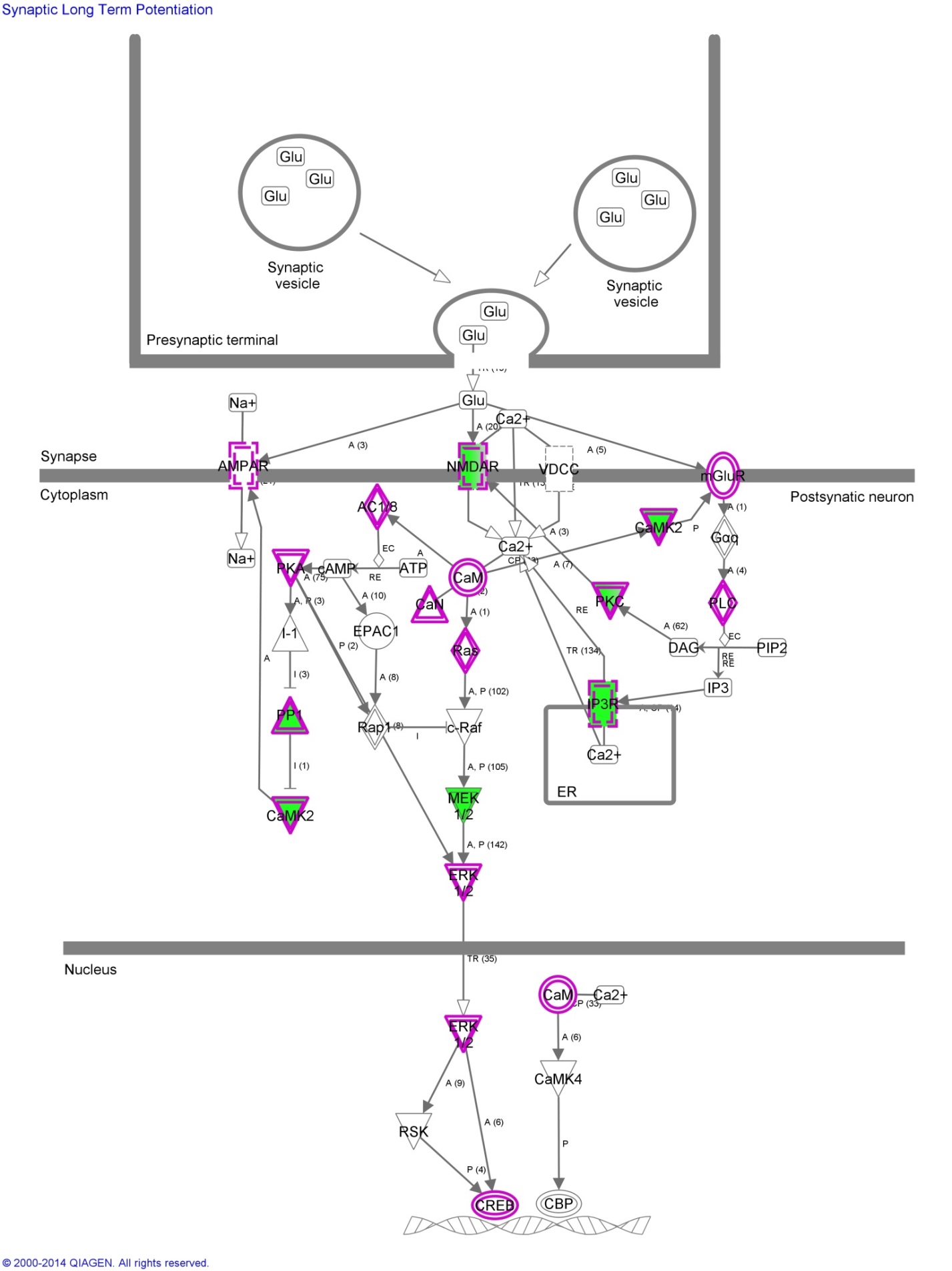


**Figure S4 :** Long term potentiation pathway is predicted to be specifically targeted by *miR-146a* and *miR-221*. Proteins in pink are predicted targets of miRNA, proteins in green are those deregulated in the post-mortem ASD brains [1]. Target prediction was performed using mirDIP and pathway enrichment analysis was performed using IPA.

| Patients | | | | | Controls | |
| --- | --- | --- | --- | --- | --- | --- |
| Code | **Age** | **Sex** | **Diagnosis** | **Principal Symptoms** | **Code** | **Age** |
| A1 | 37 | M | Infantile autism/  Severe autism  Important dependence | No verbal expression, very poor social interactions, restricted interests and activities, passivity | **C3** | 39 |
|  |  |  |  |  |  |  |
| A2 | 22 | F | Infantile autism/  Severe autism  Important dependence | Poor verbal expression (some words and sentences), poor social interactions, social avoidance, restricted interests and activities, aimless motor activity, motor stereotypes, tantrums | **C1** | 21 |
|  |  |  |  |  |  |  |
| A3 | 35 | M | Infantile autism/  Severe autism  Important dependence | Very poor verbal expression (few words), social avoidance and retrieval, restricted interests, self-injury | **C6** | 35 |
|  |  |  |  |  |  |  |
|  |  |  |  |  |  |  |
| A5 | 22 | F | Infantile autism/  Very severe autism  Complete dependence | No major verbal delay, poor gaze contact, inappropriate social interactions, cognitive disorders (dyslexia, dyscalculia), social and professional adaptation impairments | **C9** | 34 |
| A6 | 22 | M | Infantile autism/  Moderate to severe autism  Important dependence | No verbal expression, relatively good verbal comprehension, good visuo-spatial abilities, poor social interactions, motor stereotypes, aggressive behaviours | **C5** | 18 |
|  |  |  |  |  |  |  |
| A7 | 22 | M | Infantile autism/  Moderate to severe autism  Important dependence | Very poor verbal expression (few words), poor social interactions, restricted interests and activities, gestural stereotypes | **C7** | 18 |
| A8 | 43 | M | Severe autism  Important dependence | No verbal expression, relatively good verbal comprehension, poor social interactions, body sway, some aggressive behaviours | **C8** | 44 |
| A9 | 22 | M | Asperger syndrome/  Mild autism  Independence | No significant verbal nor intellectual delay, slow verbal flow, gaze avoidance, poor social contact, relatively good social and professional adaptation | **C11** | 32 |
| A10 | 38 | M | Infantile autism/  Profound autism  Complete dependence | No verbal expression, very restricted social interaction and activities, aimless motor activity, motor stereotypes, self-injury, incontinence | **C2** | 40 |
|  |  |  |  |  |  |  |
| A11 | 37 | F | Infantile autism/  Severe autism  Important dependence | Very poor verbal expression (few words), restricted verbal comprehension, gaze avoidance, restricted social interactions, motor stereotypes, aggressive behaviours | **C4** | 34 |

**Table S1 :** Clinical descriptions of ASD patients whose OMSCs were used in this study. This table was adapted from Supplementary Table 1 of the publication by [2] in which comprehensive description of the patients and the properties of the OMSC lines can be found.

| Patient | WES Coverage | | | Variant types | | | | | | Genes involved |
| --- | --- | --- | --- | --- | --- | --- | --- | --- | --- | --- |
|  | **Avg** | **5X** | **15X** | **Sub** | **Del** | **Ins** | **Hom** | **Het** | **Comp Het** |  |
| A1 | 25 | 95 | 71 | 101 | 2 | 0 | 2 | 101 | 1 | 102 |
| A2 | 57 | 98 | 93 | 91 | 5 | 3 | 1 | 98 | 1 | 98 |
| A3 | 35 | 97 | 84 | 100 | 1 | 1 | 1 | 101 | 1 | 104 |
| A5 | 66 | 98 | 95 | 75 | 2 | 1 | 1 | 77 | 1 | 77 |
| A6 | 34 | 97 | 82 | 50 | 0 | 0 | 1 | 49 | 0 | 50 |
| A7 | 59 | 98 | 93 | 85 | 6 | 0 | 3 | 88 | 4 | 88 |
| A8 | 23 | 94 | 68 | 75 | 3 | 1 | 2 | 77 | 0 | 80 |
| A9 | 18 | 90 | 53 | 67 | 1 | 2 | 3 | 67 | 0 | 71 |
| A10 | 34 | 97 | 82 | 75 | 3 | 1 | 1 | 78 | 0 | 80 |
| A11 | 20 | 92 | 61 | 72 | 3 | 1 | 0 | 76 | 1 | 76 |
| Abbreviation : WES, whole exome analysis ; Avg, average ; Sub, substitution ; Del, deletion ; Ins, insertion ; Hom, homozygous ; Het, heterozygous ; Comp Het, compound heterozygous | | | | | | | | | | |

**Table S2 :** Variants identified by whole exome analysis.

| Patient | Variants in ASD genes | | Chromosomal abnormalies | | | |
| --- | --- | --- | --- | --- | --- | --- |
|  | **Missense** | **PTC** | **Loci** | **Length** | **HG19** | **Genes involved** |
| A1 | *BZRAP1, CHD8, GABRQ* |  | 22q13.3 Del | 175,904 | chr22:51,043,246-51,219,150 | *MAPK8IP2, ARSA, SHANK3, ACR, RPL23AP82, RABL2B* |
| A2 | *NRX2* |  |  |  |  |  |
| A3 | *EPHA6* | *SCN2A, FAT1* |  |  |  |  |
| A5 |  |  | 17q12 | 1,43 Mb | chr17:34,815,327-36,249,059 | Many genes. Numerous small and few large alterations in DGV. Not associated with neuro-conditions (Cooper et al.2010) |
| A6 | *EML1, KANK1, EP300* |  |  |  |  |  |
| A7 | *TTN* |  |  |  |  |  |
| A8 |  |  |  |  |  |  |
| A9 | *EXOC6, KIAA2022* |  | 11q24.1q24.2 | 2,57 Mb | chr11:122,775,892-125,352,295 | Many genes. Numerous small and few large alterations in DGV. Not associated with neuro-conditions (Cooper et al.2010) |
| A10 |  |  | 15q13.2q13.3 | 2,1 Mb | chr15:30,730,313-3,2861,767 |  |
| A11 |  |  |  |  |  |  |
| Abbrebriation : PTC, premature termination codon | | | | | | |

**Table S3 :** Genetic etiology of patients included in this analysis.

| **Primer Names** | **Sense** | **Sequence (5'->3')** | **Tm** | | **Purposes** |
| --- | --- | --- | --- | --- | --- |
| GPBP1 qPCR F | F | TCA CTT GAG GCA GAA CAC AGA | 60 | | RT-qPCR |
| GPBP1 qPCR R | R | AGC ACA TGT TTC ATC ATT TTC AC |  |  |  |
| KCNK2_qPCR F | F | AGT TGG AGA TCA GCT AGG CAC |  |  |  |
| KCNK2_qPCR R | R | TGC GAA TCT TGG TCT GAC TAA C |  |  |  |
| GRIA3 qPCR F | F | ACC ATC AGC ATA GGT GGA CTT |  |  |  |
| GRIA3 qPCR R | R | GGT TGG TGT TGT ATA ACT GCA CG |  |  |  |
| MAP1B qPCR F | F | TCC GAC ACT TAG ACC GAG TGG |  |  |  |
| MAP1B qPCR R | R | ACA TGC TGT TTA TTC CAG GCA A |  |  |  |
| UHRF1 qPCR F | F | AGG TGG TCA TGC TCA ACT ACA |  |  |  |
| UHRF1 qPCR R | R | CAC GTT GGC GTA GAG TTC CC |  |  |  |
| FMR1 qPCR F | F | ACT TAC GGC AAA TGT GTG CCA |  |  |  |
| FMR1 qPCR R | R | GCA GAC TCC GAA AGT GCA TGT |  |  |  |
| LASS2 qPCR F | F | GCT CTT CCT CAT CGT TCG ATA C |  |  |  |
| LASS2 qPCR R | R | CTT GCC ACT GGT CAG GTA GA |  |  |  |
| APAF1 qPCR F | F | GTC ACC ATA CAT GGA ATG GCA |  |  |  |
| APAF1 qPCR R | R | CTG ATC CAA CCG TGT GCA AA |  |  |  |
| FOXO3A qPCR F | F | TAA TTG GGG CTC CGG CTA ACT |  |  |  |
| FOXO3A qPCR R | R | TGC AGG TCG CTT CCT TAT TCC |  |  |  |
| APA2A qPCR F | F | GAG ATC CCT AAG GTC CTC GTA G |  |  |  |
| APA2A qPCR R | R | GTT CTT CTG TGC TAA AGT GGT GA |  |  |  |
| MAP2 qPCR F | F | CTG CTT TAC AGG GTA GCA CAA |  |  |  |
| MAP2 qPCR F | R | TTG AGT ATG GCA AAC GGT CTG |  |  |  |
| ATRX qPCR F | F | GGT CAC TGC ATG TAA CAG CGT |  |  |  |
| ATRX qPCR R | R | GGG CAC AAT TAG TGC GGA ATA A |  |  |  |
| GRIA3_3UTR_F | F | CTT CCC CTC GAG GGC ATG TGA TGA GAG GAA ATC AC | 58 | Cloning of 3'UTR into psiCheck2 | |
| GRIA3_3UTR_R | R | TGA CTA GCG GCC GCC TCC CAT AGC TTG AAG TTA GTA GCA G |  |  |  |
| MAP2_3UTR_F | F | Ctt tgg CTC GAG atg tca ctg ctg cac tcg cta ag |  |  |  |
| MAP2_3UTR_R | R | Cca ttg GCG GCC GCc ctt tct atg gta aca ggc tct ag |  |  |  |
| KCNK2_UTR_F | F | Gac tga CTC GAG cta tct gaa tgg ttt gac gcc aca c |  |  |  |
| KCNK2_UTR_R | R | Gat ccc GCG GCC GCc ttc tgc cct cac agc caa gtc |  |  |  |
| psiCheck2_hLucF | F | CAG ATG AAA TGG GTA AGT AC | 58 | | Screening for insert |
| psiCheck2_hLucR | R | AAG ACT CAT TTA GAT CCT CA |  | |  |
| miR-146a Prom F | F | CAA GAC TGC CTT GAA TGT TCAC | 58 | | Sequecing rs57095329 |
| miR-146a Prom R | R | TGG CAG TGT CAG CTT CTC TC |  |  |  |

**Table S4 :** Primers used in this study.

| **No** | **Usage** | | **Initial** | **Sex** | **Age** | **Gene** | **Mutation** | **ID Severity** | **Diagnosis** | **ASD Score** | | |
| --- | --- | --- | --- | --- | --- | --- | --- | --- | --- | --- | --- | --- |
|  |  |  |  |  |  |  |  |  |  | **Reciprocal social interaction** | **Language & communication** | **Restrictied repetitive & stereotypic behaviours & interest** |
| ASD1 | Fibro | | Ap.K. | F | 10 | *ADSL* | c.340T>C, p.Y114H + c.1253G>C, p.418W | No ID | PDD-NOS | 19 | 13 | 5 |
| ASD2 | Fibro, PBMC | | Au.K | M | 7 | *ADSL* | c.340T>C, p.Y114H + c.1253G>C, p.418W | No ID | PDD-NOS | 11 | 9 | 5 |
| ASD3 | Fibro, PBMC | | T.K. | M | 5 | *ADSL* | c.340T>C, p.Y114H + c.1253G>C, p.418W | No ID | PDD-NOS | 11 | 9 | 5 |
| ASD4 | Fibro, PBMC | | W.S. | M | 13 | Not known | NA | No ID | Autism | 27 | 21 | 5 |
| ASD5 | Fibro | | P.G. | M | 41 | Not known | NA | No ID | Asperger | 19 | 8 | 3 |
| ASD6 | PBMC | | I. J. | M | 18 | Not known | NA | Severe | Autism | 28 | 14 | 6 |
| ASD7 | PBMC | | C.F. | F | 12 | Not known | NA | Mild | Autism | 22 | 12 | 4 |
| ASD8 | PBMC | | R.L. | M | 13 | Not known | NA | Severe | Autism | 24 | 14 | 7 |
| ASD9 | PBMC | | D.B. | M | 13 | Not known | NA | Severe | Autism | 23 | 12 | 5 |
| ASD10 | PBMC | | O.A. | M | 12 | Not known | NA | Moderate | Autism | 25 | 11 | 4 |
| ASD11 | PBMC | | A.B. | M | 12 | Not known | NA | No ID | Autism | 20 | 21 | 6 |
|  | | **Abbreviation :** ID, intellectual disability ; M, male ; F, female ; PDD-NOS, pervasive developmental disorder not otherwise specified ; NA, not applicable | | | | | | | | | | |

**Table S5:** Brief clinical descriptions of ASD patients whose primary skin fibroblasts and/or PBMC were used for follow up studies.

| **No** | **Initial** | **Sex** | **Gene** | **Mutation** | **Reference** | **No. in Reference** | **ID Severity** | **Brief clinical description** |
| --- | --- | --- | --- | --- | --- | --- | --- | --- |
| ID1 | B.C. | M | *FMR2* | CGG expansion, loss of *FMR2* expression | [3] | Fam 3, II-1 | Mild | Non-syndromic |
| ID2 | B.F. | F | *MED23* | c.1850G>A, p.R617Q | [4] | M23/R617Q | Moderate | Non-syndromic |
| ID3 | B.K. | M | *TRAPPC9* | c.1708C>T, p.R570* | [5] | Patient V-1 | Severe | Mild microcephaly, truncular obesity, hyperelorism, mild dysmorphic features, white matter abnormalities |
| ID4 | J.A. | M | *MED12* | c.3884G>A, p.R1295H | [6] | Patient MED12 | Mild | Long thin habitus, pectus excavatum, joint laxity, malar hypoplasia, downslating palpebral fissures, teeth malposition, typical Lujan Fryns Syndrome diagnosis |
| ID5 | V.G. | M | Known |  | Vasnier et al.  (manuscript in submission) | IV-12 | Severe | Axial hypotonia, tremor, eyelid myoclonia, strabismus, nystagmus, buccal breathing, cortical and subcortical atrophy, thin corpus callosum |
| ID6 | J.M. | M | *AP4B1* | c.487_488insTAT, p.E163_S739delinsV | [7] | Fam ID01 | Severe | Microcephaly, muscular hypertonia, hyperreflexia, spastic paraplegia, an inability to walk unaided, high palate, mildly remarkable facial, gestalt with a wide nasal bridge, short stature, hyperlaxity, genu recurvatum, pes planus, and a waddling gait |
| ID7 | P.A. | M | *NONO* | c.1131G>A; p.A377A | Langouët et al. (manuscript in revision) | MCCID2 | Severe | Global developmental delay, slender built, kyphoscoliosis, pesplanus, long narrow thorax. Thick corpus callosum, asymetric trigone, lateral ventricles and Chiari malformation type I |
| ID8 | M.S. | M | *HERC1* | c.9748C>T, p.Arg3250* | [8] | Patient 1 | Severe | Macrocephaly, severe myopia, seizures, no speech |
| ID9 | M.Y. | M | *AP4M1*  */AZGP1* | c.1137+1G>T | [9] | Patient II.1 | Severe | No Speech, microcephaly, epilepsy, cerebral atrophy and early onset severe obesity |
| ID10 | H.H. | F | Known |  | Not yet published |  | Severe | Mild dysmorphic features, prominent metopic |
| ID11 | C.J | M | *MECP2* | 8.6Mb Xq27.3-q28 (*MECP2*) duplication | [10] | Patient 1 | Severe | Mild dysmorphic features, cryptorchidism, bilateral pyramidal syndrome, speech delay, white matter abnormalities |
| ID12 | E.O. | M | *TTI2* | c.1307T>A, p.I436N | [9] | III-2 | Severe | Microcephaly, behavioral troubles, short stature, skeletal anomalies, and facial dysmorphic features |
| **Abbreviation :** ID, intellectual disability ; M, male ; F, female | | | | | | | | |

**Table S6 :** Brief clinical descriptions of patients with ID whose primary skin fibroblasts were used in follow up study.

| **Enriched pathways** | **-log (P Value)** | **-log (B-H p-value)** | **Ratio** | **Genes invovled** |
| --- | --- | --- | --- | --- |
| Axonal Guidance Signaling | 5,3E00 | 3,10 | 0,15 | *PRKACB,KLC1,RAC2,WNT3,MAPK1,FZD3,PIK3R1,ARPC5,CXCL12,ADAM11,SEMA4F,PTK2,SEMA6D,PAK1,PPP3R1,UNC5D,GNG12,ATM,ACTR2,AKT2,KALRN,CRKL,PLCL2,DOCK1,SDCBP,ADAMTS6,RHOA,PRKACA,PAK7,FZD5,PDGFD,ADAM22,NTF3,ARHGEF7,SEMA6A,EPHA4,FZD1,ABLIM1,ROBO1,SEMA4C,EFNB2,NFAT5,IGF1,SRGAP1,SDC2,MKNK1,PRKCE,SEMA3B,SHANK2,NRAS,NRP2,PRKAR2A,PLCG1,NFATC4,EFNA1,GNAI2,GNAI3,WIPF1,NTRK2,PRKCI,TUBA1A,WAS,PAK2,SEMA3C* |
| IL-8 Signaling | 4,25E00 | 2,41 | 0,171 | *MAP2K4,RAC2,MAPK1,PIK3R1,DIRAS3,IQGAP1,ITGB3,BCL2,IRAK1,PTK2,RHOT1,PRKCE,GNG12,EGFR,ATM,RND2,NOX4,AKT2,NRAS,GNAI2,TRAF6,FOS,GNAI3,CDH1,MPO,PRKCI,CCND2,RHOA,PAK2,MAPK10,PTGS2,KDR* |
| Signaling by Rho Family GTPases | 3,97E00 | 2,37 | 0,16 | *MAP2K4,MAPK1,PIK3R1,DIRAS3,ARHGEF7,ARPC5,PIKFYVE,PIP5K1B,IQGAP1,CDH11,PTK2,MAP3K10,PAK1,STMN1,PPP1R12B,RHOT1,GNG12,ATM,RND2,ACTR2,ARHGEF4,NOX4,CDH6,ARHGEF17,GNAI2,GNAI3,FOS,WIPF1,CDH1,PRKCI,WAS,RHOA,CDH10,PAK2,MAPK10,PAK7,MSN* |
| CXCR4 Signaling | 3,97E00 | 2,37 | 0,18 | *MAP2K4,MAPK1,PIK3R1,DIRAS3,CD4,CXCL12,PTK2,PAK1,RHOT1,PRKCE,GNG12,ATM,RND2,AKT2,NRAS,ITPR2,EGR1,GNAI2,GNAI3,DOCK1,ADCY9,FOS,PRKCI,RHOA,ADCY1,PAK2,MAPK10,PAK7* |
| Actin Cytoskeleton Signaling | 3,55E00 | 2,12 | 0,15 | *RAC2,FN1,MAPK1,PIK3R1,ARHGEF7,ARPC5,PIKFYVE,PIP5K1B,IQGAP1,SSH1,PTK2,PAK1,PPP1R12B,CYFIP2,FLNA,SSH2,GNG12,IQGAP3,ATM,ACTR2,ARHGEF4,TIAM1,NRAS,CRKL,BRK1,TTN,DOCK1,WAS,RHOA,PAK2,PAK7,PDGFD,FGF5,MSN* |
| Macropinocytosis | 2,96E00 | 1,78 | 0,21 | *NRAS,PIK3R1,USP6NL,ANKFY1,PLCG1,ITGB8,ITGB3,ARF6,PAK1,PRKCI,RHOA,PRKCE,PDGFD,ATM* |
| Synaptic Long Term Potentiation | 2,81E00 | 1,74 | 0,17 | *PRKACB,GRIN2B,NRAS,MAPK1,ITPR2,GRM1,PRKAR2A,PLCG1,PPP1R11,PLCL2,GRIA4,ATF2,CALM1,PRKCI,CAMK2D,PPP1R10,PPP3R1,ADCY1,PRKACA,PRKCE,GRIA3* |
| Netrin Signaling | 2,59E00 | 1,62 | 0,23 | *PRKACB,RAC2,NFAT5,PPP3R1,UNC5D,PRKAR2A,PRKACA,NFATC4,ABLIM1,ENAH* |
| Semaphorin Signaling in Neurons | 2,47E00 | 1,55 | 0,21 | *RND2,PTK2,PAK1,MAPK1,RHOT1,DPYSL3,DPYSL4,DIRAS3,RHOA,PAK2,PAK7* |

**Table S7 :** Selected enriched pathways predicted to be targeted by *miR-146a* and *miR-221*. Only predicted targets of *miR-146a* and/or *miR-221* by at least 3 different prediction programs (miR-DIP) were used for enrichment analysis by Ingenuiety Pathway Analysis. P Values were calculated by Fisher exact test and corrected by Bonferroni correction.

**References**

1. Voineagu I, Wang X, Johnston P, Lowe JK, Tian Y, Horvath S et al. Transcriptomic analysis of autistic brain reveals convergent molecular pathology. Nature. 2011;474(7351):380-4. doi:10.1038/nature10110.

2. Feron F, Gepner B, Lacassagne E, Stephan D, Mesnage B, Blanchard MP et al. Olfactory stem cells reveal MOCOS as a new player in autism spectrum disorders. Mol Psychiatry. 2015. doi:10.1038/mp.2015.106.

3. Gecz J, Oostra BA, Hockey A, Carbonell P, Turner G, Haan EA et al. FMR2 expression in families with FRAXE mental retardation. Hum Mol Genet. 1997;6(3):435-41. doi:dda049 [pii].

4. Hashimoto S, Boissel S, Zarhrate M, Rio M, Munnich A, Egly JM et al. MED23 mutation links intellectual disability to dysregulation of immediate early gene expression. Science. 2011;333(6046):1161-3. doi:333/6046/1161 [pii] 10.1126/science.1206638.

5. Philippe O, Rio M, Carioux A, Plaza JM, Guigue P, Molinari F et al. Combination of linkage mapping and microarray-expression analysis identifies NF-kappaB signaling defect as a cause of autosomal-recessive mental retardation. Am J Hum Genet. 2009;85(6):903-8. doi:10.1016/j.ajhg.2009.11.007.

6. Callier P, Aral B, Hanna N, Lambert S, Dindy H, Ragon C et al. Systematic molecular and cytogenetic screening of 100 patients with marfanoid syndromes and intellectual disability. Clin Genet. 2013;84(6):507-21. doi:10.1111/cge.12094.

7. Abou Jamra R, Philippe O, Raas-Rothschild A, Eck SH, Graf E, Buchert R et al. Adaptor protein complex 4 deficiency causes severe autosomal-recessive intellectual disability, progressive spastic paraplegia, shy character, and short stature. Am J Hum Genet. 2011;88(6):788-95. doi:10.1016/j.ajhg.2011.04.019.

8. Nguyen LS, Schneider T, Rio M, Moutton S, Siquier-Pernet K, Verny F et al. A nonsense variant in HERC1 is associated with intellectual disability, megalencephaly, thick corpus callosum and cerebellar atrophy. Eur J Hum Genet. 2015;In press.

9. Langouet M, Saadi A, Rieunier G, Moutton S, Siquier-Pernet K, Fernet M et al. Mutation in TTI2 reveals a role for triple T complex in human brain development. Hum Mutat. 2013;34(11):1472-6. doi:10.1002/humu.22399.

10. Philippe O, Rio M, Malan V, Van Esch H, Baujat G, Bahi-Buisson N et al. NF-kappaB signalling requirement for brain myelin formation is shown by genotype/MRI phenotype correlations in patients with Xq28 duplications. Eur J Hum Genet. 2013;21(2):195-9. doi:10.1038/ejhg.2012.140.
